# Glial- and Neuronal-Specific Expression of CCL5 mRNA in the Rat Brain

**DOI:** 10.3389/fnana.2017.00137

**Published:** 2018-01-12

**Authors:** Maria Fe Lanfranco, Italo Mocchetti, Mark P. Burns, Sonia Villapol

**Affiliations:** ^1^Laboratory of Preclinical Neurobiology, Georgetown University Medical Center, Washington, DC, United States; ^2^Department of Neuroscience, Georgetown University Medical Center, Washington, DC, United States

**Keywords:** astrocytes, corpus callosum, cerebral cortex, dopamine, hippocampus, *in situ* hybridization, oligodendrocytes, ventral tegmental area

## Abstract

Chemokine (C-C motif) ligand 5 (CCL5) belongs to a group of chemokines that play a role in the peripheral immune system, mostly as chemoattractant molecules, and mediate tactile allodynia. In the central nervous system (CNS), CCL5 and its receptors have multiple functions, including promoting neuroinflammation, insulin signaling, neuromodulator of synaptic activity and neuroprotection against a variety of neurotoxins. Evidence has also suggested that this chemokine may regulate opioid response. The multifunctional profile of CCL5 might correlate with its ability to bind different chemokine receptors, as well as with its unique cellular expression. In this work, we have used fluorescence *in situ* hybridization combined with immunohistochemistry to examine the expression profile of CCL5 mRNA in the adult rat brain and provide evidence of its cellular localization. We have observed that the highest expression of CCL5 mRNA occurs in all major fiber tracts, including the corpus callosum, anterior commissure, and cerebral peduncle. In these tracts, CCL5 mRNA was localized in oligodendrocytes, astrocytes and microglia. Astrocytic and microglial expression was also evident in several brain areas including the cerebral cortex, caudate/putamen, hippocampus, and thalamus. Furthermore, using a specific neuronal marker, we observed CCL5 mRNA expression in discrete layers of the cortex and hippocampus. Interestingly, in the midbrain, CCL5 mRNA co-localized with tyrosine hydroxylase (TH) positive cells of the ventral tegmental area, suggesting that CCL5 might be expressed by a subset of dopaminergic neurons of the mesolimbic system. The expression of CCL5 mRNA and protein, together with its receptors, in selected brain cell populations proposes that this chemokine could be involved in neuronal/glial communication.

## Introduction

Chemokines belong to the cytokine family of peptides that induce the maturation and trafficking of leukocytes and are considered to be essential for the inflammatory responses of the immune system. Some chemokines have been linked to pro-inflammatory events, associated with chronic pain (White et al., 2005) or brain diseases such as human immunodeficiency virus (HIV)-associated dementia (Schmidtmayerova et al., 1996; Conant et al., 1998), multiple sclerosis (Hvas et al., 1997; McManus et al., 1998; Rentzos et al., 2007), stroke (Siniscalchi et al., 2014) or traumatic brain injury (Villapol et al., 2017).

Of particular interest is the chemokine (C-C motif) ligand 5 (CCL5), formerly known as Regulated on Activation Normal T-cell Expressed and Secreted (RANTES). CCL5 is a small protein of 68 amino acids that activate mononuclear phagocytes and induce their migration across the blood brain barrier to the site of inflammation (Ubogu et al., 2006). The role of CCL5 in inflammation has also been inferred by an association between increased CCL5 protein expression and the degree of inflammation in a variety of disorders and pathologies, including neuropathic pain (Bhangoo et al., 2007), asthma, atherosclerosis and arthritis among others (Marques et al., 2013). However, in the brain, CCL5 function goes beyond the one attributed to a classic pro-inflammatory chemokine. In fact, CCL5 is capable of inducing proliferation of Oli-*neu*, an oligodendrocyte precursor-like cell line (Kadi et al., 2006) suggesting a role in myelination. Moreover, CCL5 promotes the migration of dorsal root ganglia cells *in vitro* (Bolin et al., 1998), and regulates the differentiation of astrocytes (Bakhiet et al., 2001) suggesting that CCL5 may act as a neurotrophic factor. In addition, *ex vivo* activation of CCL5 receptor (CCR5) by CCL5 increases glucose transporter type 4 membrane translocation in the hypothalamus (Chou et al., 2016), suggesting a role of CCL5 in glucose uptake and metabolism. These examples highlight the potential role of CCL5 as a modulator of cellular metabolism and brain architecture. Lastly, CCL5 exerts neuroprotective activity against various neurotoxins including glutamate (Bruno et al., 2000), β-amyloid (Ignatov et al., 2006), and the HIV proteins gp120 (Campbell et al., 2015) and tat (Rozzi et al., 2014). The different effects of CCL5 could be due to the ability of this chemokine to bind to multiple receptors. The selective and differential expression of CCL5 and its receptors, CCR5 (Avdoshina et al., 2011), CCR3 (He et al., 1997) and CCR1 (Tran et al., 2007) in the rodent brain supports the role of CCL5 as a potential modulator of brain homeostasis. Nevertheless, the functional role of CCL5 in the brain could be more in line with the suggested properties of some chemokines to act as a third neurotransmitter system (Adler et al., 2005) helping neuronal communication (Rostene et al., 2007), perhaps through modulation of the release of glutamate from nerve endings (Musante et al., 2008).

CCL5 is constitutively expressed in the adult central nervous system (CNS; Campbell et al., 2013). Nevertheless, the type of cells that express CCL5 has been so far inferred by *in vitro* studies. For instance, astrocytes appear to express CCL5 at high levels (Avdoshina et al., 2010); nevertheless, oligodendrocytes (Balabanov et al., 2007), microglia (Avdoshina et al., 2010) and neurons (Mocchetti et al., 2013), all release CCL5. In this study, we have analyzed CCL5 mRNA expression patterns in the rat brain using *in situ* hybridization combined with immunohistochemistry to detect specific cell types (Grabinski et al., 2015; Lanfranco et al., 2017). This method showed adequate sensitivity and specificity to detect mRNA transcripts of the CCL5 gene. Our results show that CCL5 mRNA follows a cellular and anatomical distribution, which is highly regionalized and restricted to certain parts of the brain. We provide evidence for the first time that CCL5 mRNAs can be expressed in dopamine-producing neurons.

## Materials and Methods

### Animals

Two month-old C57BL/6J (wild-type, WT) and CCL5 knock-out (KO, B6.129P2-Ccl5tm1) male mice (22–25 g) were purchased from The Jackson Laboratory (Bar Harbor, ME, USA). CCL5 KO mice have been validated by northern blot analysis of total RNA isolated from LPS-stimulated peritoneal exudate cells (Makino et al., 2002). Three-month-old male Sprague–Dawley (SD) rats (225–250 g) were purchased from Charles River Laboratory (Germantown, MD, USA). Animals were housed under standard conditions with food and water *ad libitum* and maintained on a 12-h light/dark cycle. Animals were anesthetized with a mixture of ketamine/xylazine (80 mg/kg and 10 mg/kg, i.p.) and intracardially perfused with ice-cold phosphate buffered saline (PBS) followed by perfusion with 4% paraformaldehyde (PFA). After perfusion, whole brains were quickly removed and post-fixed in 4% PFA overnight and transferred sequentially into a 10%, 20% and 30% sucrose solution. Post-fixed brains were used for RNA *in situ* hybridization and immunohistochemistry studies. All studies were carried out following the Guide for the Care and Use of Laboratory Animals as adopted and promulgated by the U.S. National Institutes of Health and approved by Georgetown University Animal Care and Use Committee.

### RNAscope^®^ *in Situ* Hybridization Combined with Immunohistochemistry

A sliding microtome (microm HM 430, Thermo Fisher Scientific, Tustin, CA, USA) was used to cut the brains in coronal orientation. The brain sections (20 μm thickness) were cryoprotected in an antifreeze solution (30% glycerol + 30% ethylene glycol + 0.01 M PBS) for storage at −20°C. Free-floating brain sections were washed three times in PBS before mounting in Gold Seal™ UltraStick™ Adhesion Microscope Slides (Cat No. 3039-002, Thermo Fisher Scientific). Tissue was allowed to dry at room temperature (RT) and then stored at −20°C until use. RNAscope^®^ *in situ* hybridization assay was performed according to manufacturer’s instructions (Advance Cell Diagnostics (ACD), Hayward, CA, USA). In short, mounted tissue sections were serially dehydrated in 50%, 70%, 95%, 100% and 100% ethanol for 5 min each. In between all pretreatment steps, tissue sections were briefly washed with ultra-pure water. Incubation periods were performed on the HybEz™ hybridization system (ACD). The pretreat solution 1 (hydrogen peroxide reagent) was applied for 10 min at RT and then the tissue sections were boiled in pretreat solution 2 (target retrieval reagent) for 15 min. Mounted slices were treated with pretreat solution 3 (protease reagent) for 30 min at 40°C. Custom rat CCL5 RNAscope^®^ probe was designed and purchased from ACD. CCL5 probe targets the region 14-556 (Accession number: NM_031116.3) of the CCL5 sequence with 12 pairs of ZZ-target probes. In addition, the negative (Cat. No. 310043, ACD) and positive (Cat. No. 313911, ACD) control probes were applied and let hybridized for 2 h at 40°C. The amplification steps were performed according to manufacturer’s directions. In between every amplification step, sections were washed with 1× wash buffer. Detection was performed using a mixture ratio of Red-A to Red-B solution of 1:60. The sections were incubated for 10 min at RT and rinsed with ultra-pure water.

Following *in situ* hybridization, the sections were processed for immunohistochemistry. Briefly, following the blocking step with 5% normal goat serum (NGS) in PBS for 1 h at RT, post-hybridized slides were incubated with an antibody against glial fibrillary acidic protein (GFAP, 1:1000, EMD Millipore, Temecula, CA, USA), ionized calcium binding adaptor molecule-1 (Iba-1, 1:500, Wako Chemical USA, Richmond, VA, USA), homeobox protein transcription factor Nkx2.2 (1:100, EMD Millipore), neuronal nuclear antigen (NeuN, 1:100, EMD Millipore) or tyrosine hydroxylase (TH, 1:500, EMD Millipore) in the presence of 2% NGS in PBS overnight at 4°C. Brain slices treated with NeuN antibody were incubated for 72 h at 4°C. Subsequent to three washes with PBS, the slides were incubated with corresponding Alexa Fluor^®^ 488 secondary antibodies (1:500; Molecular probes^®^, Thermo Fisher Scientific) for 2 h at RT. Brain sections were rinsed with PBS three times and incubated for 5 min in PBS with DAPI solution (1:50,000, Sigma-Aldrich, St. Louis, MO, USA) for counterstained nuclei.

### Images Acquisition and Quantitative Analysis

Anatomical structures were analyzed in coronal sections and mapped according to Paxinos and Watson atlas (Paxinos and Watson, 1998). Fluorescent signals of CCL5 mRNA hybridization and immunohistochemistry for different cell types were imaged with a 10×, 20×, 40× and 63× objective lens on a Leica SP8 confocal microscope (Leica Microsystems Inc., Buffalo Grove, IL, USA) and an Axioplan 2 microscope (Zeiss, Thorwood, NY, USA) with a Photometrics camera. All microscope and camera settings were identical for all images. The color label to far red (Excitation 647 nm, Emission 690 nm) was assigned for mRNA hybridization signal. The color label to green was assigned for the antigen of interest (Excitation 490 nm, Emission 525 nm). The number of CCL5 mRNA positive/cell type marker, and the total number of CCL5 mRNA positive cells were quantified in five (corpus callosum and cortex) and three (hippocampus and VTA) microscopic fields per brain section (20X, 151.894 mm^2^), using the ImageJ64 software (National Institute of Health. Bethesda, MD, USA), as previously described (Villapol et al., 2017).

### Statistical Analysis

Figures for RNAscope and IHC are representative of sections obtained from three animals. Data for Figure 4, expressed as the mean ± SEM (sections obtained from five animals), were analyzed using one-way analysis of variance (ANOVA) with Bonferroni’s multiple comparison *post hoc* test using GraphPad Prism software v.5.0 (Graphpad). A *p*-value < 0.05 was considered statistically significant.

**Figure 1:**
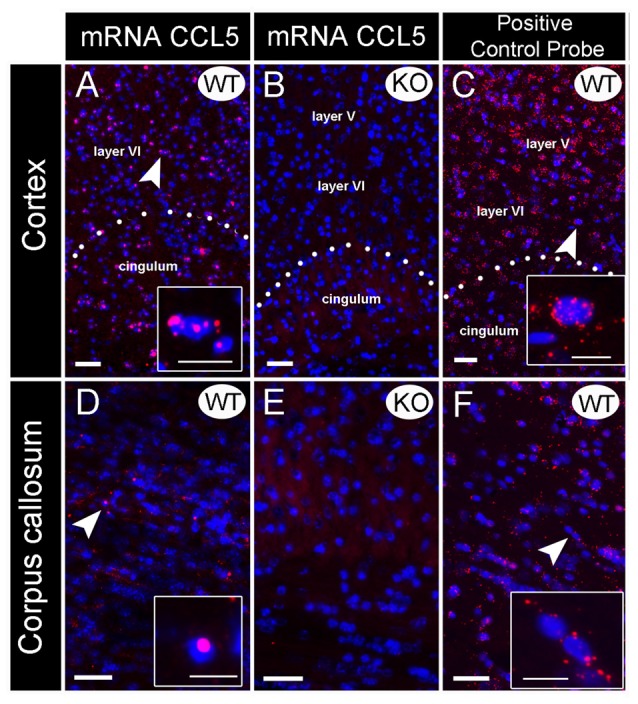
Validation of CCL5 mRNA probe using CCL5 knock-out (KO) mice. *In situ* hybridization was performed with an RNAscope probe targeting CCL5 mRNA. Representative images for CCL5 mRNA (red dots) and nuclei counterstained with DAPI (blue) in the cortex **(A–C)** and corpus callosum **(D–F)** of wild-type (WT) **(A,D)** and KO **(B,E)** mice. The sections were also analyzed for peptidylprolyl isomerase B mRNA **(C,F)** as positive control probe. *Insets*: high magnification images depicted by the white arrows. Images showed that the fluorescent *in situ* hybridization by the RNAscope probe produces puncta (red dots) that represents a single mRNA transcript. Scale bar 50 μm for **(A–F)** and 20 μm for inset images.

**Figure 2:**
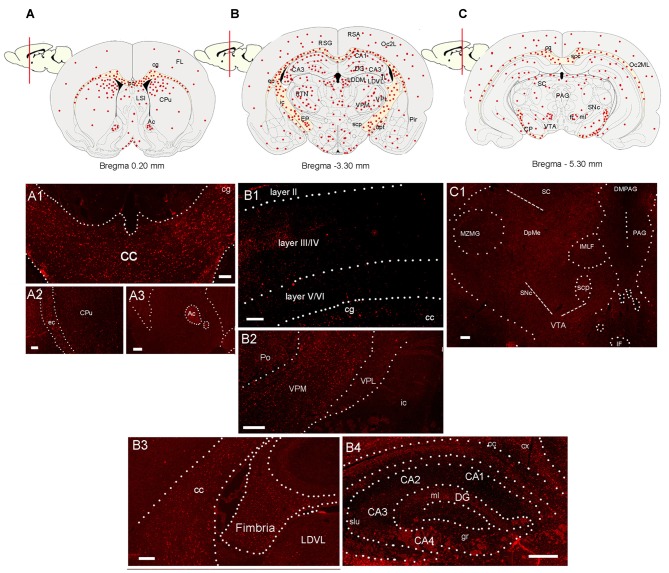
Distribution of CCL5 mRNA positive cells in the rat brain. **(A–C)** Schematic drawing of three coronal sections of the rat brain indicating where CCL5 mRNA puncta (red dots) were detected. **(A1–A3)** Representative sections through the corpus callosum (cc) and cingulate cortex (cg), external capsule (ec) and caudate/putamen (CPu), and anterior commissure (Ac). **(B1)** Representative section through the cerebral cortex denoting CCL5 mRNA punta in different cortical layers (II-IV). **(B2)** Representative section through the thalamus; ic, internal capsule; Po, posterior thalamic nucleus; VPM, ventroposterior medial nucleus; VPL, ventroposterior lateral nucleus; **(B3,B4)** representative sections through the hippocampal formation; LDVL, laterodorsal tegmental nucleus; slu, stratum lucidum of the hippocampus; gr, gracile fasciculus; ml, medial lemniscus; cx, parietal cortex; DG, dentate gyrus; (CA1-4), Cornu Ammonis 1-4. **(C1)** Representative section from the midbrain; scp, superior cerebellar peduncle; SNc, *substantia nigra* compacta; MZMG, marginal zone of the medial geniculate nucleus; sc, superior colliculus; DpME, deep mesencephalic nucleus; PAG, periaqueductal gray; DMPAG, dorsomedial PAG; IF, interfascicular nucleus; VTA, ventral tegmental area. Scale bar 50 μm for **(A1–C1)**.

**Figure 3:**
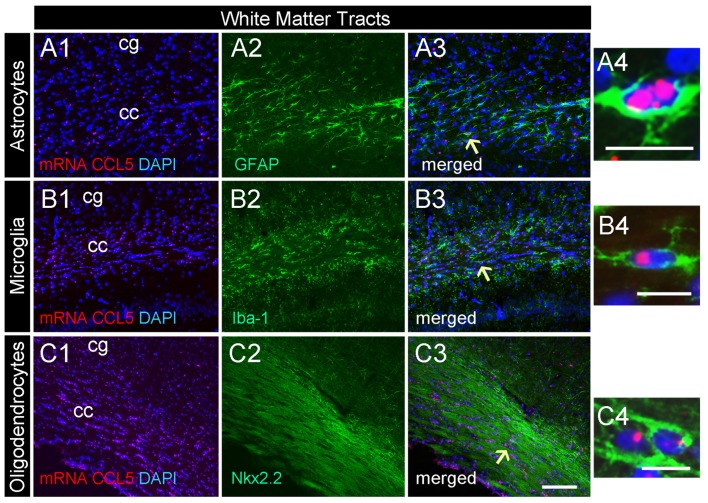
CCL5 mRNA in the corpus callosum is expressed by glia cells. Representative images of coronal sections showing the distribution of red dots in the corpus callosum. Positive CCL5 mRNA hybridization was observed in GFAP **(A1–3)**, Iba-1 **(B1–3)**, and Nkx2.2 **(C1–3)** positive cells. Panels **(A4–C4)** are higher magnifications of the areas shown by arrows. DAPI (blue) was used as a counterstaining to show nuclei. cc, corpus callosum; cg, cingulate gyrus. Scale bar = 50 μm for **(A1–3,B1–3,C1–3)**; 20 μm for **(A4–C4)**.

**Figure 4:**
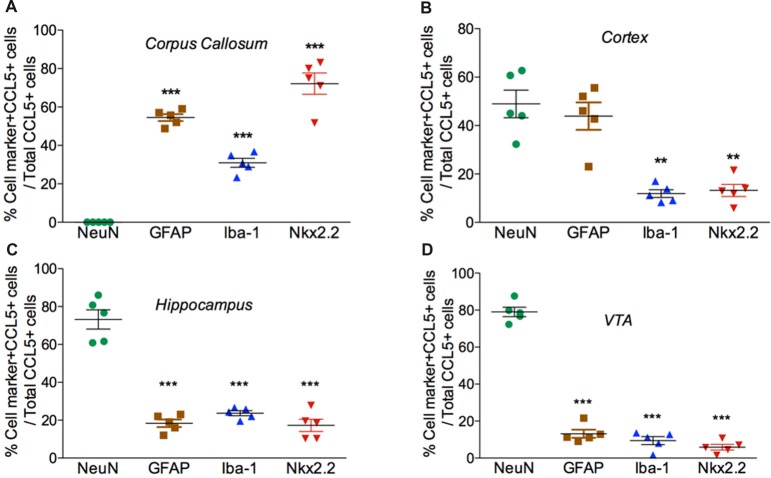
Cell expression of CCL5 mRNA follows an anatomical profile. Quantifications of CCL5 mRNA puncta within cells were performed on a series of five randomly selected coronal sections per rat, throughout the corpus callosum **(A)**, cerebral cortex **(B)**, hippocampus **(C)**, and VTA **(D)**. Data, represented as the mean ± SEM, are expressed as the average of double-stained cells compared to the total number of CCL5 mRNA positive cells per animal (*n* = 5). **p < 0.01; ***p < 0.001 neurons vs. glial cell types.

## Results

### Validation of CCL5 mRNA Probe

Several antibodies against CCL5 exhibit non-specific binding to an antigen as determined by positive reactions in brain sections from WT and CCL5 KO mice (Supplementary Figure S1). Thus, we used *in situ* hybridization to map the expression of CCL5 mRNA. Rat and mouse CCL5 mRNA share high sequence similarity (>90%). Therefore, we first validated the specificity of the CCL5 probe for RNAscope^®^ by comparing CCL5 mRNA signal in brain sections from WT and CCL5 KO mice. In the cortex (Figure 1A) and corpus callosum (Figure 1D) of WT mice, the CCL5 probe produced a strong fluorescent signal, characterized by red dots co-localizing with DAPI positive cells (insets in Figures 1A,D), suggesting a nuclear localization. Moreover, we observed that only a subpopulation of cells expresses CCL5 mRNA, evidenced by the lack of red puncta in several DAPI-stained nuclei (Figures 1A,D). No hybridization was observed in CCL5 KO mice (Figures 1B,E), supporting the specificity of the CCL5 mRNA probe used for RNAscope^®^.

Additional controls for potential artifacts were performed. Sections were stained with a positive control probe that targeted the peptidylprolyl isomerase B gene. This gene is ubiquitously expressed throughout the mice tissue (Kouadjo et al., 2007). In a parallel experiment, sections were stained with a probe that targeted the *Bacillus subtilis* dihydrodipicolinate reductase gene. This was used as a negative control because this bacterial gene is not expressed in mice. While peptidylprolyl isomerase B probe detected an mRNA species throughout the brain (Figures 1C,F), the negative control probe generated no fluorescence (data not shown) suggesting that the technique and conditions used do not create artifacts.

### Detection of CCL5 mRNA in the Rat Brain

RNAscope^®^ was then used to map the expression of CCL5 mRNA throughout the adult rat brain. Figures 2A–C show a schematic representation of three representative coronal brain sections (0.20 mm, −3.30 mm and −5.30 mm from Bregma) where CCL5 mRNA was detected. Puncta were observed throughout the brain, although some regions contained more puncta than others (Table 1). For instance, the corpus callosum (cc, Figure 2A1) and the anterior commissure (Ac, Figure 2A3) contained more puncta than the caudate/putamen (CPu, Figure 2A2). In the cerebral cortex, more hybridization was observed in layers III/IV than II and VI (Figure 2B1). In the thalamus, the ventroposterior medial (VPM) nucleus exhibited more CCL5 mRNA hybridization than the ventroposterior lateral (VPL) nucleus (Figure 2B2). Within the hippocampus, CCL5 mRNA was more abundant in the fornix than in the hippocampal formation (Figures 2B3,B4). In the midbrain, areas with CCL5 mRNA signal included the cerebral peduncles (CP, Figure 2C), and the ventral tegmental area (VTA, Figure 2C1).

**Table 1:** Relative levels of CCL5 mRNA.

Brain regions	CCL5 mRNA
**White matter tracts**	
Corpus callosum	++++
External capsule	++++
Internal capsule	+++
Fasciculus retroflexus	++++
Anterior commissure	++++
Medial lemniscus	++
Optic tract	+++
**Cerebral cortex**	
Forelimb area	+
Frontal cortex	++
Parietal cortex	++
Occipital cortex	++
Cingulate cortex	++++
Piriform cortex	+
Retrosplenial dysgranular cortex (RSG)	++
Retrosplenial granular cortex (RSA)	++
**Septal nuclei/Caudate**	
Lateral septal nucleus	++
Vertical limb of diagonal band	+++
Dorsal caudate/putamen	+++
Nucleus accumbens	+
Entopeduncular nucleus	++
**Hippocampus/Amygdala**	
Fimbria hippocampus (fi)	++++
CA1	++
CA2	+
CA3	++
Dentate gyrus (DG)	+++
Amygdala	+
**Thalamus/Hypothalamus**	
Laterodorsal thalamus, ventrolateral (LDVL)	++
Ventral posterolateral thalamic nucleus (VPL)	++
Ventral posteromedial thalamic nucleus (VPM)	+++
Hypothalamus	++
**Midbrain**	
Superior colliculus	+
Cerebral peduncle	++
Superior cerebellar peduncle/red nucleus	+++
Substantia nigra pars compacta	+
Substantia nigra pars reticulata	++
Medial pretectal nucleus	+++
Ventral tegmental area (VTA)	++
Periaqueductal gray	+

*The levels of CCL5 mRNA are arbitrarily graded as occasional (+), low (++), moderate (+++), and high (++++) based on the intensity of hybridization signal*.

Semi-quantitative analyses revealed that CCL5 mRNA is expressed in several brain regions, in agreement with previous studies (Campbell et al., 2013), but that there is an overall higher expression of CCL5 mRNA in areas containing fiber tracts. These include the corpus callosum, anterior commissure, external capsule, fimbria, optic tract and cerebral peduncle (Table 1). Moreover, we observed CCL5 mRNA positive signal in several brain areas, including the cerebral cortex, striatum, hippocampus, thalamus and midbrain (Table 1). Within these brain structures, CCL5 mRNA was detected only in selected areas. For instance, the piriform cortex was negative (Table 1). In the striatum, the dorsomedial portion exhibited more CCL5 mRNA signal than the ventromedial one (Table 1). Taken together, our data suggest that different cell type may express CCL5 mRNA.

### Glial Expression of CCL5 mRNA in the Corpus Callosum

Our semi-quantitative analysis indicates that CCL5 mRNA is most abundant in the white matter that contains fiber tracts (Table 1) which includes several subtypes of glial cells. To reveal which cells express CCL5 mRNA, we examined the corpus callosum utilizing RNAscope^®^ *in situ* hybridization combined with immunohistochemistry for specific glial cell markers. These include GFAP, an astrocytic marker, Iba-1, a microglia/macrophage-specific marker, and Nkx2.2, a marker to identify oligodendrocyte progenitor cells. We found that CCL5 mRNA in the corpus callosum is predominantly inside nuclei of cells, evidenced by the co-localization of DAPI (blue) with the CCL5 mRNA hybridization signal, that were in GFAP (Figures 3A1–4), Iba-1 (Figures 3B1–4) and Nkx2.2 (Figures 3C1–4) positive cells. Noteworthy, Nkx2.2 positive cells were aligned in parallel rows (Figure 3C3), which is a typical property of adult oligodendrocyte progenitor cells. Quantitative analyses of CCL5 mRNA in the corpus callosum revealed that 53.3%, 31.4% and 72.4% of GFAP, Iba-1 and Nkx2.2 positive cells, respectively, expressed CCL5 mRNA while no CCL5 mRNA was found in neurons (Figure 4A). These results validate the notion that the corpus callosum contains a high percentage of oligodendrocytes expressing CCL5 mRNA.

### CCL5 mRNA Subcellular Distribution in the Cerebral Cortex

**Figure 5:**
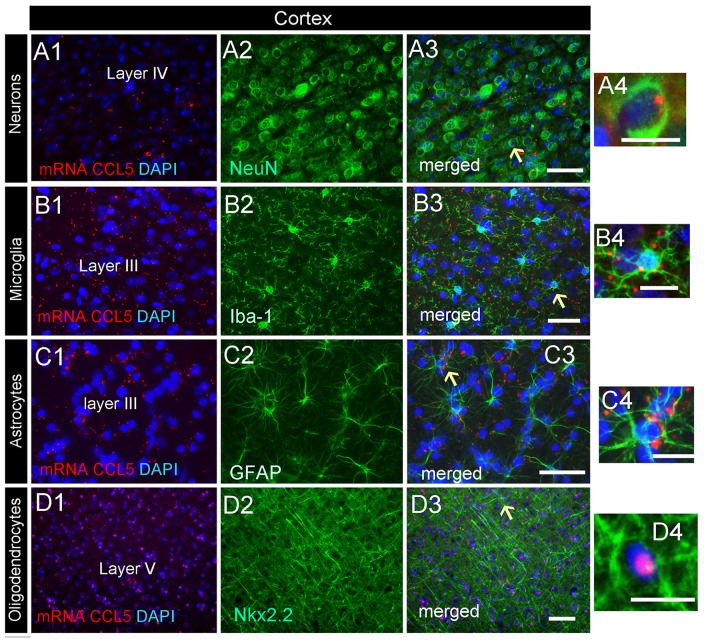
Cell-specific localization of CCL5 mRNA in the cerebral cortex. RNAscope and immunohistochemistry were used to identify cells within the primary somatosensory cortex expressing CCL5 mRNA. **(A1–3)** Representative images of coronal sections showing the localization of CCL5 mRNA (red dots) in NeuN positive cells (green). Nuclei were counterstained with DAPI (blue). CCL5 mRNA (red dots) was also co-localized with Iba-1 **(B1–3)** and GFAP **(C1–3)** positive cells (green). Panels **(D1–3)** are examples of CCL5 mRNA signal (red dots) in Nkx2.2 (green) positive cells in layer V. Panels **(A4–D4)** are higher magnifications of the areas indicated by the arrows. Scale bar = 50 μm for **(A1–D3)**; 20 μm for high magnification images **(A4–D4)**.

Analysis of the somatosensory cortex with the CCL5 probe revealed that layers III-V contained CCL5 mRNA, although most of the puncta were in layers III and IV (Figure 2B1). To characterize which cells express CCL5 mRNA in these layers, serial sections were examined by RNAscope^®^ *in situ* hybridization combined with immunohistochemistry, using glial specific markers as described above. The neuron-specific nuclear protein marker, NeuN, was used to detect neurons. We observed that the fluorescent puncta of CCL5 mRNAs in layers III and IV were co-localized with NeuN (Figures 5A1–4), Iba-1 (Figures 5B1–4), as well as GFAP (Figures 5C1–4) positive cells. More positive puncta were observed in glial cells (Figures 5B4,C4) than neurons (Figure 5A4), suggesting that cortical neurons express CCL5 mRNA in low abundance. Although we did not quantify CCL5 protein levels due to the lack of specificity of the antibodies, this result supports previous *in vitro* data that neurons produce and release CCL5 protein at a lower level than glial cells (Avdoshina et al., 2010). In layer V, CCL5 mRNA was also observed in Nkx2.2 positive cells (Figures 5D1–4), overall suggesting that in the cerebral cortex glia cell as well neurons express CCL5 mRNA. To confirm this finding, we carried out a quantitative analysis of CCL5 mRNA puncta, 48.9% of neurons, 44.1% of astrocytes, 12% of microglia and 13.3% oligodendrocytes were also positive for CCL5 mRNA (Figure 4B).

### Neurons in the Hippocampus Express CCL5 mRNA

**Figure 6:**
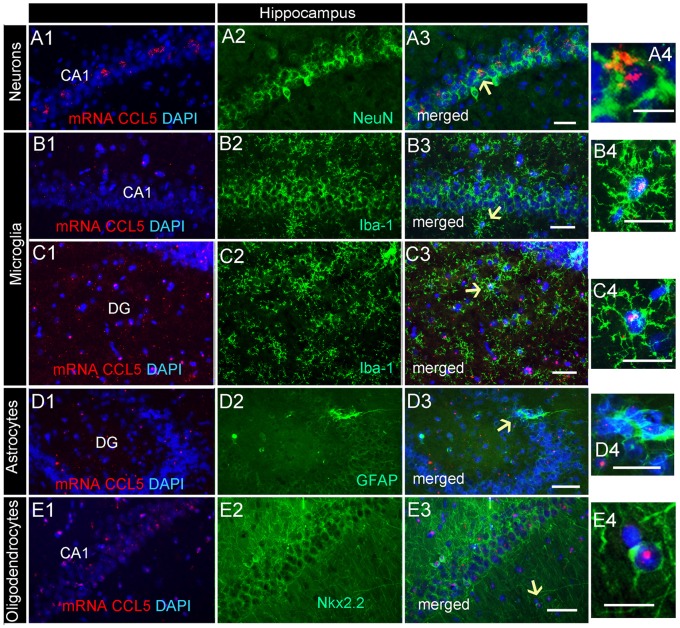
Distribution of CCL5 mRNA in the hippocampus. Images are representative coronal sections showing the distribution of CCL5 mRNA (red dots) in the dentate gyrus (DG) and CA1 regions of the hippocampus. The sections were stained for NeuN **(A1–3)**, Iba-1 **(B1–3,C1–3)**, GFAP **(D1–3)**, or Nkx2.2 **(E1–3)**. DAPI (blue) was used as a counterstaining. Panels **(A4–E4)** are higher magnifications of areas pointed by arrows. Scale bar = 50 μm for **(A1–E3)**, and 20 μm for high magnification images **(A4–E4)**.

Analysis of the hippocampal formation revealed that CCL5 mRNA is particularly abundant in the fimbria (Figure 2B3), which is a prominent white matter tract along the edge of the hippocampus, supporting the notion that CCL5 mRNA is expressed in fiber tracts. However, red puncta were also observed in sections throughout the hippocampus mainly in the *Cornu Ammonis* (CA)1 and dentate gyrus (DG) regions (Figure 2B4). The CA1 contains pyramidal neurons whereas the polymorphic layer of the DG consists of sparsely distributed polymorphic cells. RNAscope^®^ *in situ* hybridization combined with immunohistochemistry revealed that in the CA1 region, in addition to neurons (Figures 6A1–4), microglia (Figures 6B1–4) and oligodendrocytes (Figures 6E1–4) expressed CCL5 mRNA. In neurons, the red puncta that correspond to CCL5 mRNA were found outside and inside the nuclei (Figure 6A4), suggesting that some of this mRNA is readily available for translation. In the polymorphic cells layer of the DG, microglia (Figures 6C1–4) and astrocytes (Figures 6D1–4) were positive for CCL5 mRNA. Overall, in the hippocampus, CCL5 mRNA was found in 73.2% of neurons and in ~20% in glial cells (Figure 4C).

### CCL5 mRNA Is Expressed in a Subset of Dopaminergic Neurons in the Midbrain

**Figure 7:**
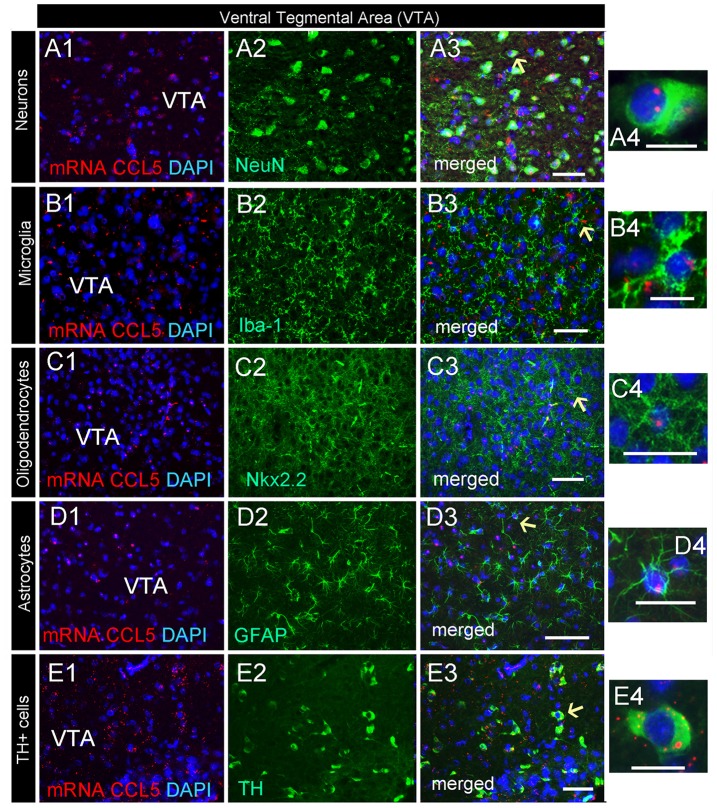
Dopamine neurons express CCL5 mRNA. Representative images of coronal sections through the VTA. CCL5 mRNA (red dots) was detected in DAPI (blue) positive nuclei of neurons **(A1–3)**, microglia **(B1–3)**, oligodendrocytes **(C1–3)** and astrocytes **(D1–3)**. CCL5 mRNA is also expressed in tyrosine hydroxylase (TH) positive cells **(E1–3)**. Panels **(A4–E4)** are higher magnifications of areas pointed by arrows. Scale bar = 50 μm for **(A1–E3)**, and 20 μm for high magnification images **(A4–E4)**.

CCL5 mRNA was also scattered in the midbrain (Figure 2C1). The majority of puncta were observed in the cerebral peduncle (Figure 2C), a major descending fiber tract. However, puncta were also observed within the VTA region. RNAscope^®^ *in situ* hybridization followed by immunohistochemistry revealed that in this region, 79% of neurons (Figure 4D) express CCL5 mRNA (Figures 7A1–4). Microglia (Figures 4D, 7B1–4), astrocytes (Figures 7D1–4), and few oligodendrocytes (Figures 4D, 7C1–4) also express CCL5 mRNA in 11.4%, 13.7%, and 5.9% respectively. Intriguingly, only a subpopulation of NeuN positive cells expressed CCL5 mRNA (Figure 7A3). To determine the type of neurons that express CCL5 mRNA, sections were stained with an antibody against TH, the rate limiting step enzyme for the synthesis of dopamine. Some but not all TH-positive cells exhibited CCL5 mRNA puncta (Figures 7E1–3), suggesting that CCL5 mRNA is synthesized in a small subset of dopamine neurons. Interestingly, these neurons, unlike those in the cerebral cortex, exhibited several puncta, mostly perinuclearly (Figure 7E4), suggesting high expression of this mRNA in these neurons.

## Discussion

Our results reveal a unique expression profile of CCL5 mRNA throughout the adult rodent brain. We showed that the cellular anatomical distribution of this chemokine is highly regionalized and restricted to certain parts of the brain. Most notably, we found that CCL5 mRNA hybridization signal is the highest in white matter, such as the anterior commissure, corpus callosum and the optic tract, which are rich in glia cells and myelinated axons. However, a constitutive expression of CCL5 mRNA was also present in some subset of neuronal cell in the cerebral cortex and hippocampus, and especially in dopamine-producing neurons of the VTA. Altogether, our data characterize a constitutively anatomical and cellular expression of CCL5 mRNA in the rat brain. Most importantly, we observed CCL5 mRNA in various cells even without injury or inflammation.

Our work joins a growing number of studies that have shown that chemokines can be constitutively expressed throughout the brain in a region specific manner. For example, CX3CL1, the first chemokine shown to be expressed in neurons, is constitutively expressed in human (Raport et al., 1995) and rat brain (Nishiyori et al., 1998). CXCL12, also known as stromal derived factor-1, is expressed in different cell types throughout the rat brain, including astrocytes, microglia, and neurons (Banisadr et al., 2003). Similarly, CCL2 is constitutively expressed in neurons and astrocytes, but not in oligodendrocytes or microglia (Banisadr et al., 2005). Our data shows that under physiological conditions CCL5 mRNA is constitutively expressed in all glial cells (microglia, astrocytes and oligodendrocytes) as well as in a subset of neurons. Although in this work we only measured mRNA, the fact that CCL5 proteins are detected in the adult brain (Campbell et al., 2013) supports the notion that CCL5, like other chemokines (Banisadr et al., 2003), might have a role as potential modulators of brain function.

Under physiological conditions, CCL5 is expressed in low abundance in the brain tissue, posing an experimental challenge to detect it with conventional immunostaining technology. In addition, levels of a given peptide do not necessarily reflect the site of synthesis. An alternative approach is measuring mRNA by RNAscope^®^ technology, which in addition to the sensitivity for the detection of low abundance mRNA molecules (Sørdal et al., 2013). In our study, we were able to observe low number of puncta within cells, as well as an agglomerate of puncta. The highest hybridization signal was mostly observed in glial cells. Moreover, in several sections, we have seen puncta in DAPI positive cells, suggesting a nuclear localization. However, CCL5 mRNA was also localized outside the nucleus. Thus, it appears that from the nucleus, CCL5 mRNA might move to the cytoplasm, where the translation into CCL5 protein occurs. This event could be followed by CCL5 release. Although measuring CCL5 release *in vivo* is technically challenging, this notion is supported by previous data showing that brain cells *in vitro* release CCL5 proteins (Avdoshina et al., 2010).

A novel discovery presented here is the expression of CCL5 mRNA in oligodendrocytes. The significance of this result is still under investigation. CCL5 promotes the proliferation of an oligodendrocyte precursor-like cell line (Kadi et al., 2006). Thus, it is possible that oligodendrocyte precursors or other glial cells might be regulating their own proliferation via the release of CCL5. Oligodendrocyte most well known role is to preserve myelin; however, oligodendrocytes also maintain axonal integrity, support axonal metabolism and aid neuronal survival (Peferoen et al., 2014). Changes in oligodendrocytes number and dynamics are increasingly recognized as important components in the pathogenesis of neurodegenerative disorders. In *taiep* rats, an animal model for multiple sclerosis, CCL5 and its receptor CCR5 is down-regulated in comparison to Sprague-Dawley rats (Soto-Rodriguez et al., 2015). Such reduction has been shown to limit the infiltration of pro-inflammatory macrophages and therefore to prevent demyelination (Glass et al., 2001). On the other hand, CCL5 has neuroprotective properties. For example, CCL5 has been shown to exert neuroprotective activity against various neurotoxins including glutamate (Bruno et al., 2000), β-amyloid (Ignatov et al., 2006), and the viral proteins gp120 (Campbell et al., 2015) and Tat (Rozzi et al., 2014). The neuroprotective activity of CCL5 could be due to its ability to increase neurotrophic factors, such as brain-derived neurotrophic factor and epidermal growth factor (Tokami et al., 2013). Thus, it is conceivable to suggest that oligodendrocyte-derived CCL5 could act as a regulator of neuronal survival.

Our studies showed that some neuronal populations in selected brain areas exhibit CCL5 mRNA. CCL5 is not the only chemokine that is expressed in neurons. For example, CCL2 is constitutively expressed in neurons, and co-localizes with various neurotransmitters and neuropeptides (Banisadr et al., 2005). Also, CXCL12, CXCL14 and CX3CL1 are expressed in neurons in several brain regions (Nishiyori et al., 1998; Banisadr et al., 2011). In addition, neurons, at least in culture, are capable of releasing CCL5 upon depolarization or activation of N-methyl-D-aspartate receptors (Mocchetti et al., 2013), suggesting that neurons, under the proper stimulation, are capable of releasing CCL5, not dissimilar to that observed for neurotransmitters. Here, we show that NeuN positive cells in the cerebral cortex and in the hippocampus are also CCL5 mRNA positive; together with previous findings that cortical neurons release CCL5 (Avdoshina et al., 2010), our data suggest that CCL5 mRNA can be translated into proteins in neurons. Moreover, few TH-positive cells in the VTA expressed CCL5 mRNA, indicating that CCL5 may be expressed by a subset of dopaminergic neurons. Why CCL5 mRNA is detected only a subset of dopamine neurons is at present unclear. The VTA also contains GABAergic neurons (Dobi et al., 2010). Thus, the co-localization of CCL5 mRNA in TH-negative but NeuN positive cells in the VTA suggests that CCL5 mRNA may be synthetized in GABAergic neurons. More experiments are needed to support this suggestion.

Our data show that CCL5 mRNA is contained in a subset of dopaminergic neurons. The significance of this finding remains speculative at present. Dopamine neurons of the VTA belong to the dopaminergic mesolimbic system, which comprises several interconnected brain regions, including the nucleus accumbens and the prefrontal cortex. Activation of this system is crucial for the reward and addictive properties of opioids (Wise and Rompre, 1989; Pontieri et al., 1995). We have previously shown that CCL5 protein levels are increased in morphine dependent rats in brain areas involved in opioid reward (Campbell et al., 2013). CCL5 has also been shown to interact with opioid receptors causing heterologous desensitization (Szabo et al., 2002). Thus, CCL5, when produced in dopaminergic fibers innervating the nucleus accumbens or prefrontal cortex could be contributing to regulation of dependence to opioids as previously suggested (Campbell et al., 2013). CCL5 might act as a neuromodulator to enhance/inhibit the synaptic transmission underlying opioid reward. Although still speculative, this suggestion is supported by data showing that CCR5, the receptor for CCL5, is expressed in selected neuronal populations of the frontal cortex and striatum (Avdoshina et al., 2011). Moreover, CCL5 increases the release of glutamate (Musante et al., 2008; Di Prisco et al., 2012) whose activity could induce various forms of synaptic plasticity and cellular adaptations that are responsible for opioid tolerance and addiction (Manzoni and Williams, 1999; Stuber et al., 2010, 2011).

In conclusion, we were able to detect CCL5 mRNA by *in situ* hybridization in adult rat brain in several cells, including a subset of neurons. Together with previous results showing that CCL5 and its receptor CCR5 are constitutively produced in the adult rodent brain (Tran et al., 2007; Avdoshina et al., 2011; Campbell et al., 2013), our data suggest a role for CCL5 in the CNS other than a homeostatic chemokine that attracts and activates mononuclear phagocytes at sites of inflammation.

## Author Contributions

MFL, IM and SV designed the experiments and wrote the manuscript. MFL and SV performed the *in situ* hybridization and immunofluorescence experiments and analyzed the data. IM, SV and MPB revised the manuscript. All authors reviewed the results and approved the final version of the manuscript.

## Conflict of Interest Statement

The authors declare that the research was conducted in the absence of any commercial or financial relationships that could be construed as a potential conflict of interest.

## Supplementary Material

FIGURE S1 Examples of brain sections from wild-type (WT) and CCL5 knock-out (KO) mice stained for CCL5 using different commercially available antibodies. **(A,B)** 25H14L17 (1:2000, Thermo Fisher), **(C,D)** NBP1-19769 (1:500, Novus Biological), **(E,F)** LS-C104689 (1:1500, LifeSpan BioSciences, Inc.), **(G,H)** SAB2107938 (1:500, Sigma-Aldrich). Please note that all antibodies detect CCL5-like immunoreactivity in KO mice. At higher dilutions, all antibodies failed to detect CCL5 immunoreactivity.

## References

[B1] Adler M. W. Geller E. B. Chen X. Rogers T. J. (2005). Viewing chemokines as a third major system of communication in the brain. AAPS J. 7, E865–E870. 10.1208/aapsj070484 16594639 PMC2750956

[B2] Avdoshina V. Becker J. Campbell L. Parsadanian M. Mhyre T. Tessarollo L.. (2011). Neurotrophins modulate the expression of chemokine receptors in the brain. J. Neurovirol. 17, 58–62. 10.1007/s13365-010-0004-3 21165786 PMC3077968

[B3] Avdoshina V. Biggio F. Palchik G. Campbell L. A. Mocchetti I. (2010). Morphine induces the release of CCL5 from astrocytes: potential neuroprotective mechanism against the HIV protein gp120. Glia 58, 1630–1639. 10.1002/glia.21035 20578038 PMC2919594

[B4] Bakhiet M. Tjernlund A. Mousa A. Gad A. Strömblad S. Kuziel W. A.. (2001). RANTES promotes growth and survival of human first-trimester forebrain astrocytes. Nat. Cell Biol. 3, 150–157. 10.1038/35055057 11175747

[B5] Balabanov R. Strand K. Goswami R. McMahon E. Begolka W. Miller S. D.. (2007). Interferon-γ-oligodendrocyte interactions in the regulation of experimental autoimmune encephalomyelitis. J. Neurosci. 27, 2013–2024. 10.1523/JNEUROSCI.4689-06.2007 17314297 PMC6673565

[B6] Banisadr G. Bhattacharyya B. J. Belmadani A. Izen S. C. Ren D. Tran P. B.. (2011). The chemokine BRAK/CXCL14 regulates synaptic transmission in the adult mouse dentate gyrus stem cell niche. J. Neurochem. 119, 1173–1182. 10.1111/j.1471-4159.2011.07509.x 21955359 PMC3330702

[B7] Banisadr G. Gosselin R. D. Mechighel P. Kitabgi P. Rostene W. Parsadaniantz S. M. (2005). Highly regionalized neuronal expression of monocyte chemoattractant protein-1 (MCP-1/CCL2) in rat brain: evidence for its colocalization with neurotransmitters and neuropeptides. J. Comp. Neurol. 489, 275–292. 10.1002/cne.20598 16025454

[B8] Banisadr G. Skrzydelski D. Kitabgi P. Rostène W. Parsadaniantz S. M. (2003). Highly regionalized distribution of stromal cell-derived factor-1/CXCL12 in adult rat brain: constitutive expression in cholinergic, dopaminergic and vasopressinergic neurons. Eur. J. Neurosci. 18, 1593–1606. 10.1046/j.1460-9568.2003.02893.x 14511338

[B9] Bhangoo S. Ren D. Miller R. J. Henry K. J. Lineswala J. Hamdouchi C.. (2007). Delayed functional expression of neuronal chemokine receptors following focal nerve demyelination in the rat: a mechanism for the development of chronic sensitization of peripheral nociceptors. Mol. Pain 3:38. 10.1186/1744-8069-3-38 18076762 PMC2228278

[B10] Bolin L. M. Murray R. Lukacs N. W. Strieter R. M. Kunkel S. L. Schall T. J.. (1998). Primary sensory neurons migrate in response to the chemokine RANTES. J. Neuroimmunol. 81, 49–57. 10.1016/s0165-5728(97)00158-6 9521605

[B11] Bruno V. Copani A. Besong G. Scoto G. Nicoletti F. (2000). Neuroprotective activity of chemokines against N-methyl-D-aspartate or β-amyloid-induced toxicity in culture. Eur. J. Pharmacol. 399, 117–121. 10.1016/s0014-2999(00)00367-8 10884510

[B12] Campbell L. A. Avdoshina V. Day C. Lim S. T. Mocchetti I. (2015). Pharmacological induction of CCL5 *in vivo* prevents gp120-mediated neuronal injury. Neuropharmacology 92, 98–107. 10.1016/j.neuropharm.2015.01.009 25623966 PMC4346538

[B13] Campbell L. A. Avdoshina V. Rozzi S. Mocchetti I. (2013). CCL5 and cytokine expression in the rat brain: differential modulation by chronic morphine and morphine withdrawal. Brain Behav. Immun. 34, 130–140. 10.1016/j.bbi.2013.08.006 23968971 PMC3795805

[B14] Chou S. Y. Ajoy R. Changou C. A. Hsieh Y. T. Wang Y. K. Hoffer B. (2016). CCL5/RANTES contributes to hypothalamic insulin signaling for systemic insulin responsiveness through CCR5. Sci. Rep. 6:37659. 10.1038/srep37659 27898058 PMC5127185

[B15] Conant K. Garzino-Demo A. Nath A. McArthur J. C. Halliday W. Power C.. (1998). Induction of monocyte chemoattractant protein-1 in HIV-1 Tat-stimulated astrocytes and elevation in AIDS dementia. Proc. Natl. Acad. Sci. U S A 95, 3117–3121. 10.1073/pnas.95.6.3117 9501225 PMC19704

[B16] Di Prisco S. Summa M. Chellakudam V. Rossi P. I. Pittaluga A. (2012). RANTES-mediated control of excitatory amino acid release in mouse spinal cord. J. Neurochem. 121, 428–437. 10.1111/j.1471-4159.2012.07720.x 22385043

[B17] Dobi A. Margolis E. B. Wang H.-L. Harvey B. K. Morales M. (2010). Glutamatergic and nonglutamatergic neurons of the ventral tegmental area establish local synaptic contacts with dopaminergic and nondopaminergic neurons. J. Neurosci. 30, 218–229. 10.1523/jneurosci.3884-09.2010 20053904 PMC3209506

[B18] Glass W. G. Liu M. T. Kuziel W. A. Lane T. E. (2001). Reduced macrophage infiltration and demyelination in mice lacking the chemokine receptor CCR5 following infection with a neurotropic coronavirus. Virology 288, 8–17. 10.1006/viro.2001.1050 11543653 PMC7142305

[B19] Grabinski T. M. Kneynsberg A. Manfredsson F. P. Kanaan N. M. (2015). A method for combining RNAscope *in situ* hybridization with immunohistochemistry in thick free-floating brain sections and primary neuronal cultures. PLoS One 10:e0120120. 10.1371/journal.pone.0120120 25794171 PMC4368734

[B20] He J. Chen Y. Farzan M. Choe H. Ohagen A. Gartner S.. (1997). CCR3 and CCR5 are co-receptors for HIV-1 infection of microglia. Nature 385, 645–649. 10.1038/385645a0 9024664

[B21] Hvas J. McLean C. Justesen J. Kannourakis G. Steinman L. Oksenberg J. R.. (1997). Perivascular T cells express the pro-inflammatory chemokine RANTES mRNA in multiple sclerosis lesions. Scand. J. Immunol. 46, 195–203. 10.1046/j.1365-3083.1997.d01-100.x 9584001

[B22] Ignatov A. Robert J. Gregory-Evans C. Schaller H. C. (2006). RANTES stimulates Ca^2+^ mobilization and inositol trisphosphate (IP_3_) formation in cells transfected with G protein-coupled receptor 75. Br. J. Pharmacol. 149, 490–497. 10.1038/sj.bjp.0706909 17001303 PMC2014681

[B23] Kadi L. Selvaraju R. de Lys P. Proudfoot A. E. Wells T. N. Boschert U. (2006). Differential effects of chemokines on oligodendrocyte precursor proliferation and myelin formation *in vitro*. J. Neuroimmunol. 174, 133–146. 10.1016/j.jneuroim.2006.01.011 16574247

[B24] Kouadjo K. E. Nishida Y. Cadrin-Girard J. F. Yoshioka M. St-Amand J. (2007). Housekeeping and tissue-specific genes in mouse tissues. BMC Genomics 8:127. 10.1186/1471-2164-8-127 17519037 PMC1888706

[B200] Lanfranco M. F. Loane D. J. Mocchetti I. Burns M. P. Villapol S. (2017). Combination of fluorescent *in situ* hybridization (FISH) and immunofluorescence imaging for detection of cytokine expression in microglia/macrophage cells. Bio Protoc. 7:e2608 10.21769/BioProtoc.2608 PMC5725199 29238736

[B25] Makino Y. Cook D. N. Smithies O. Hwang O. Y. Neilson E. G. Turka L. A.. (2002). Impaired T cell function in RANTES-deficient mice. Clin. Immunol. 102, 302–309. 10.1006/clim.2001.5178 11890717

[B26] Manzoni O. J. Williams J. T. (1999). Presynaptic regulation of glutamate release in the ventral tegmental area during morphine withdrawal. J. Neurosci. 19, 6629–6636. 10414991 10.1523/JNEUROSCI.19-15-06629.1999 PMC6782799

[B27] Marques R. E. Guabiraba R. Russo R. C. Teixeira M. M. (2013). Targeting CCL5 in inflammation. Expert Opin. Ther. Targets 17, 1439–1460. 10.1517/14728222.2013.837886 24090198 PMC7103722

[B28] McManus C. Berman J. W. Brett F. M. Staunton H. Farrell M. Brosnan C. F. (1998). MCP-1, MCP-2 and MCP-3 expression in multiple sclerosis lesions: an immunohistochemical and *in situ* hybridization study. J. Neuroimmunol. 86, 20–29. 10.1016/s0165-5728(98)00002-2 9655469

[B29] Mocchetti I. Campbell L. A. Harry G. J. Avdoshina V. (2013). When human immunodeficiency virus meets chemokines and microglia: neuroprotection or neurodegeneration? Brain Res. 8, 118–131. 10.1007/s11481-012-9353-4 22527632 PMC3427402

[B30] Musante V. Longordo F. Neri E. Pedrazzi M. Kalfas F. Severi P.. (2008). RANTES modulates the release of glutamate in human neocortex. J. Neurosci. 28, 12231–12240. 10.1523/JNEUROSCI.3212-08.2008 19020017 PMC6671730

[B31] Nishiyori A. Minami M. Ohtani Y. Takami S. Yamamoto J. Kawaguchi N.. (1998). Localization of fractalkine and CX3CR1 mRNAs in rat brain: does fractalkine play a role in signaling from neuron to microglia? FEBS Lett. 429, 167–172. 10.1016/s0014-5793(98)00583-3 9650583

[B32] Paxinos G. Watson C. (1998). The Rat Brain in Stereotaxic Coordinates. San Diego, CA: Academic Press.

[B33] Peferoen L. Kipp M. van der Valk P. van Noort J. M. Amor S. (2014). Oligodendrocyte-microglia cross-talk in the central nervous system. Immunology 141, 302–313. 10.1111/imm.12163 23981039 PMC3930369

[B34] Pontieri F. E. Tanda G. Di Chiara G. (1995). Intravenous cocaine, morphine, and amphetamine preferentially increase extracellular dopamine in the “shell” as compared with the “core” of the rat nucleus accumbens. Proc. Natl. Acad. Sci. U S A 92, 12304–12308. 10.1073/pnas.92.26.12304 8618890 PMC40345

[B35] Raport C. J. Schweickart V. L. Eddy R. L. Jr. Shows T. B. Gray P. W. (1995). The orphan G-protein-coupled receptor-encoding gene V28 is closely related to genes for chemokine receptors and is expressed in lymphoid and neural tissues. Gene 163, 295–299. 10.1016/0378-1119(95)00336-5 7590284

[B36] Rentzos M. Nikolaou C. Rombos A. Boufidou F. Zoga M. Dimitrakopoulos A.. (2007). RANTES levels are elevated in serum and cerebrospinal fluid in patients with amyotrophic lateral sclerosis. Amyotroph. Lateral Scler. 8, 283–287. 10.1080/17482960701419232 17852013

[B37] Rostene W. Kitabgi P. Parsadaniantz S. M. (2007). Chemokines: a new class of neuromodulator? Nat. Rev. Neurosci. 8, 895–903. 10.1038/nrn2255 17948033

[B38] Rozzi S. J. Borelli G. Ryan K. Steiner J. P. Reglodi D. Mocchetti I.. (2014). PACAP27 is protective against tat-induced neurotoxicity. J. Mol. Neurosci. 54, 485–493. 10.1007/s12031-014-0273-z 24696163 PMC4185276

[B39] Schmidtmayerova H. Nottet H. S. Nuovo G. Raabe T. Flanagan C. R. Dubrovsky L.. (1996). Human immunodeficiency virus type 1 infection alters chemokine β peptide expression in human monocytes: implications for recruitment of leukocytes into brain and lymph nodes. Proc. Natl. Acad. Sci. U S A 93, 700–704. 10.1073/pnas.93.2.700 8570619 PMC40116

[B40] Siniscalchi A. Gallelli L. Malferrari G. Pirritano D. Serra R. Santangelo E.. (2014). Cerebral stroke injury: the role of cytokines and brain inflammation. J. Basic Clin. Physiol. Pharmacol. 25, 131–137. 10.1515/jbcpp-2013-0121 24515999

[B41] Sørdal Ø. Qvigstad G. Nordrum I. S. Gustafsson B. Waldum H. L. (2013). *In situ* hybridization in human and rodent tissue by the use of a new and simplified method. Appl. Immunohistochem. Mol. Morphol. 21, 185–189. 10.1097/PAI.0b013e31825a0048 22688353

[B42] Soto-Rodriguez G. Gonzalez-Barrios J. A. Martinez-Fong D. Blanco-Alvarez V. M. Eguibar J. R. Ugarte A.. (2015). Analysis of chemokines and receptors expression profile in the myelin mutant taiep rat. Oxid. Med. Cell. Longev. 2015:397310. 10.1155/2015/3973100 25883747 PMC4390177

[B43] Stuber G. D. Hnasko T. S. Britt J. P. Edwards R. H. Bonci A. (2010). Dopaminergic terminals in the nucleus accumbens but not the dorsal striatum corelease glutamate. J. Neurosci. 30, 8229–8233. 10.1523/JNEUROSCI.1754-10.2010 20554874 PMC2918390

[B44] Stuber G. D. Sparta D. R. Stamatakis A. M. van Leeuwen W. A. Hardjoprajitno J. E. Cho S.. (2011). Excitatory transmission from the amygdala to nucleus accumbens facilitates reward seeking. Nature 475, 377–380. 10.1038/nature10194 21716290 PMC3775282

[B45] Szabo I. Chen X. H. Xin L. Adler M. W. Howard O. M. Oppenheim J. J.. (2002). Heterologous desensitization of opioid receptors by chemokines inhibits chemotaxis and enhances the perception of pain. Proc. Natl. Acad. Sci. U S A 99, 10276–10281. 10.1073/pnas.102327699 12130663 PMC124904

[B46] Tokami H. Ago T. Sugimori H. Kuroda J. Awano H. Suzuki K.. (2013). RANTES has a potential to play a neuroprotective role in an autocrine/paracrine manner after ischemic stroke. Brain Res. 1517, 122–132. 10.1016/j.brainres.2013.04.022 23602964

[B47] Tran P. B. Banisadr G. Ren D. Chenn A. Miller R. J. (2007). Chemokine receptor expression by neural progenitor cells in neurogenic regions of mouse brain. J. Comp. Neurol. 500, 1007–1033. 10.1002/cne.21229 17183554 PMC2758702

[B48] Ubogu E. E. Callahan M. K. Tucky B. H. Ransohoff R. M. (2006). Determinants of CCL5-driven mononuclear cell migration across the blood–brain barrier. Implications for therapeutically modulating neuroinflammation. J. Neuroimmunol. 179, 132–144. 10.1016/j.jneuroim.2006.06.004 16857269

[B49] Villapol S. Loane D. J. Burns M. P. (2017). Sexual dimorphism in the inflammatory response to traumatic brain injury. Glia 65, 1423–1438. 10.1002/glia.23171 28608978 PMC5609840

[B50] White F. A. Bhangoo S. K. Miller R. J. (2005). Chemokines: integrators of pain and inflammation. Nat. Rev. Drug Discov. 4, 834–844. 10.1038/nrd1852 16224455 PMC2792904

[B51] Wise R. A. Rompre P. P. (1989). Brain dopamine and reward. Annu. Rev. Psychol. 40, 191–225. 10.1146/annurev.psych.40.1.191 2648975

