# Supplementary figures and images for: Glial- and Neuronal-Specific Expression of CCL5 mRNA in the Rat Brain
Source: Front Neuroanat. 2018 Jan 12;11:137. doi: 10.3389/fnana.2017.00137 (PMC5770405; doi:10.3389/fnana.2017.00137)

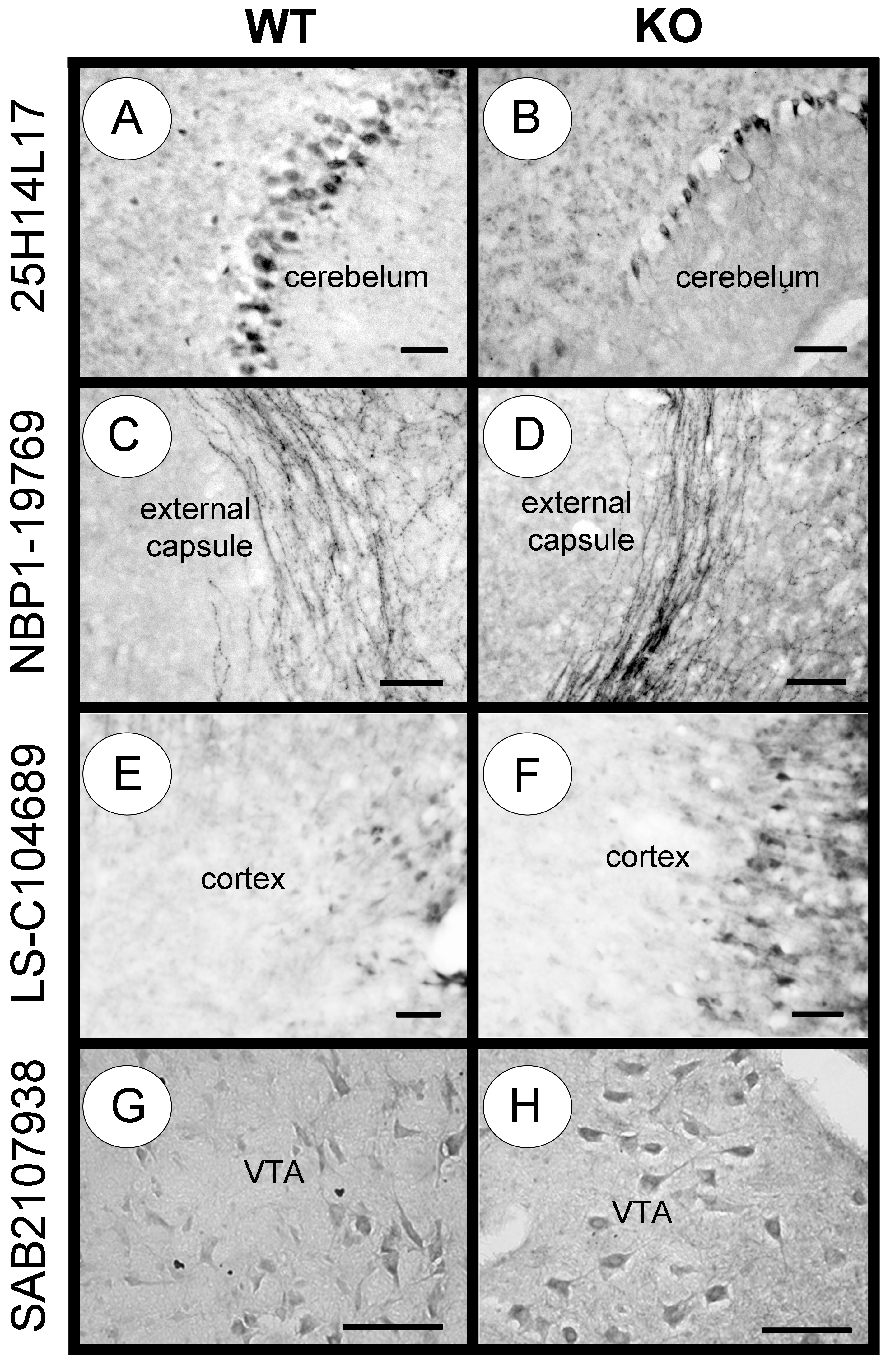

Supplement: FIGURE S1 — Examples of brain sections from wild-type (WT) and CCL5 knock-out (KO) mice stained for CCL5 using different commercially available antibodies. (A,B) 25H14L17 (1:2000, Thermo Fisher), (C,D) NBP1-19769 (1:500, Novus Biological), (E,F) LS-C104689 (1:1500, LifeSpan BioSciences, Inc.), (G,H) SAB2107938 (1:500, Sigma-Aldrich). Please note that all antibodies detect CCL5-like immunoreactivity in KO mice. At higher dilutions, all antibodies failed to detect CCL5 immunoreactivity. [file Image_1.jpeg]
